# Detection of peanut seed vigor based on hyperspectral imaging and chemometrics

**DOI:** 10.3389/fpls.2023.1127108

**Published:** 2023-02-27

**Authors:** Zhiyong Zou, Jie Chen, Weijia Wu, Jinghao Luo, Tao Long, Qingsong Wu, Qianlong Wang, Jiangbo Zhen, Yongpeng Zhao, Yuchao Wang, Yongming Chen, Man Zhou, Lijia Xu

**Affiliations:** ^1^ College of Mechanical and Electrical Engineering, Sichuan Agricultural University, Yaan, China; ^2^ School of Electrical Engineering and Automation, Hubei Normal University, Huangshi, Hubei, China; ^3^ Food Academy, Sichuan Agricultural University, Yaan, China

**Keywords:** hyperspectral imaging technology, peanut seed vitality, superoxide dismutase activity, nondestructive testing technology, data analysis

## Abstract

Rapid nondestructive testing of peanut seed vigor is of great significance in current research. Before seeds are sown, effective screening of high-quality seeds for planting is crucial to improve the quality of crop yield, and seed vitality is one of the important indicators to evaluate seed quality, which can represent the potential ability of seeds to germinate quickly and whole and grow into normal seedlings or plants. Meanwhile, the advantage of nondestructive testing technology is that the seeds themselves will not be damaged. In this study, hyperspectral technology and superoxide dismutase activity were used to detect peanut seed vigor. To investigate peanut seed vigor and predict superoxide dismutase activity, spectral characteristics of peanut seeds in the wavelength range of 400-1000 nm were analyzed. The spectral data are processed by a variety of hot spot algorithms. Spectral data were preprocessed with Savitzky-Golay (SG), multivariate scatter correction (MSC), and median filtering (MF), which can effectively to reduce the effects of baseline drift and tilt. CatBoost and Gradient Boosted Decision Tree were used for feature band extraction, the top five weights of the characteristic bands of peanut seed vigor classification are 425.48nm, 930.8nm, 965.32nm, 984.0nm, and 994.7nm. XGBoost, LightGBM, Support Vector Machine and Random Forest were used for modeling of seed vitality classification. XGBoost and partial least squares regression were used to establish superoxide dismutase activity value regression model. The results indicated that MF-CatBoost-LightGBM was the best model for peanut seed vigor classification, and the accuracy result was 90.83%. MSC-CatBoost-PLSR was the optimal regression model of superoxide dismutase activity value. The results show that the R^2^ was 0.9787 and the RMSE value was 0.0566. The results suggested that hyperspectral technology could correlate the external manifestation of effective peanut seed vigor.

## 1 Introduction

Peanut is an important oil crop mainly used for oil production, and by-products of peanut also contain many other functional compounds such as protein, fiber, polyphe-nols, antioxidants, vitamins and minerals, which can be added as functional ingredients in many processed foods (Arya et al., 2016). Factors affecting the yield and quality of peanuts include climate temperature and humidity, fertilization ratio, sowing density, degree of pests and diseases, and seed vigor of peanut seeds(Gomes et al., 2009). Among many factors, seed vigor plays a crucial role (Tu et al., 2022). If the problem of aging or vigor decline of peanut seeds during storage is not solved, it will cause delayed seed germination (Wang et al., 2021), poor growth potential, weak stress resistance, and biological Yields and economic yields are reduced, thereby affecting agricultural production. Therefore, in the production and application of peanut crops, judging the vigor of peanut seeds has a guiding role in improving the yield and quality of peanuts.

Generally speaking, the seed vigor of peanut is difficult to judge by manual observation. The appearance of aged peanut seeds is almost the same as that of fresh peanut seeds except that the gloss of the seed surface is slightly rough. Therefore, methods such as standard seed germination detection method (Zhang et al., 2020), field emergence test method (Kaya et al., 2019), conductivity measurement method (Xiang et al., 2020), TTC detection method (Liu et al., 2009), and red ink measurement method (Mattioni et al., 2015) are usually used to identify whether peanut seeds are fresh or aged. However, these methods require experienced operators, and farmers cannot master this skill well. At the same time, these methods are highly destructive, inefficient, time-consuming, and complicated in operation procedures, and are not suitable for rapid non-destructive testing of peanut seed viability. Compared with traditional destructive inspection methods, machine vision technology (Tan et al., 2022) and near-infrared spectroscopy technology (Jasinski et al., 2016) have been widely used in seed quality inspection, such as variety (Kotwaliwale et al., 2019), mildew (Fu et al., 2017), vigor (Liu et al., 2021), and defect (Wang et al., 2022). However, machine vision only uses phenotypic features, such as color, size, shape and surface texture, and is not suitable for predicting the chemical composition of a sample, because the internal chemical content of seeds changes after aging (Huang & Chien, 2017); Near-infrared spectroscopy can be used to assess the chemical composition of a sample, but it is only used to obtain spectral information from a single point of light, and is always affected by the uniformity of the sample distribution, and is not the best choice (ElMasry et al., 2019).

Composed of spatial imaging, spectroscopy (Kucha et al., 2021), and chemical measurement tools, hyperspectral imaging techniques (Xu et al., 2021) can provide information on seed quality characteristics and characterization parameters(Jun Yang et al., 2021), overcoming the limitations of machine vision and near-infrared spectroscopy techniques(Laborde et al., 2021). In recent years, some studies have used hyperspectral imaging technology as a powerful tool for seed vigor monitoring. Compared with the traditional seed vigor detection technology based on biological properties, the non-destructive seed vigor detection technology based on hyperspectral imaging technology is gradually attracting the attention of the seed industry. Some scholars only use hyperspectral imaging technology to discriminate seed vigor. Such as He Xiantao et al. used hyperspectral imaging technology to predict rice seed viability within three years, combined with various preprocessing and feature band extraction algorithms, the results showed that the classification accuracy reached 94.38% (He et al., 2019). Some studies combine hyperspectral imaging techniques(Zou et al., 2022) and texture feature methods. Such as Wang Zheli et al. used hyperspectral imaging technology and chemometrics to quickly and nondestructively classify new and old corn seeds, and established a model of spectral and image texture features, with a classification accuracy rate of 95% (Wang et al., 2022). There are also studies that integrate hyperspectral imaging technology and mapping technology. For example, Yan Lei et al. used hyperspectral imaging technology combined with machine learning and convolutional neural network to identify seed germination with an accuracy of 99.96% (Pang et al., 2020).

Previous studies have demonstrated the potential of hyperspectral imaging techniques and provided a good reference for the field of seed quality inspection. However, most of the current research stays at the macroscopic stage, and only uses hyperspectral technology to identify whether the seeds are aging or not, and has not analyzed the factors affecting the seed vigor. In fact, factors that affect seed vigor include seed moisture content, protein content, superoxide dismutase content (SOD) (Matłok et al., 2022), catalase (CAT), ascorbate peroxidase (As A-POD) and guaiacol peroxidase (G-POD) (Zhao et al., 2021). Among them, SOD is involved in catalyzing the disproportionation reaction with reactive oxygen species and free radicals as substrates, and its activity level directly affects the seed vigor (Bandeira et al., 2014). Therefore, the SOD of peanut seeds was measured and modeled in this paper.

The overall goal of this study was to examine the potential of hyperspectral imaging in the detection of aged peanut seeds using samples of varying degrees of aging, while establishing a microscopic content analysis of superoxide dismutase in peanut seeds. The specific goals are (1) To establish a hyperspectral-based classification model for peanut seed viability detection through standard roll paper germination tests; (2) To identify and evaluate the best characteristic wavelengths for peanut seed detection; (3) Determination of peanut seed SOD value by tetrazolium blue (NBT) method, and establishment of a peanut seed SOD regression model based on hyperspectral spectrum. From the perspective of spectroscopy, it is revealed that the characteristic band, seed vigor index and SOD value have strong correlation, which provides a new method for seed quality evaluation.

## 2 Materials and methods

**Figure 1:**
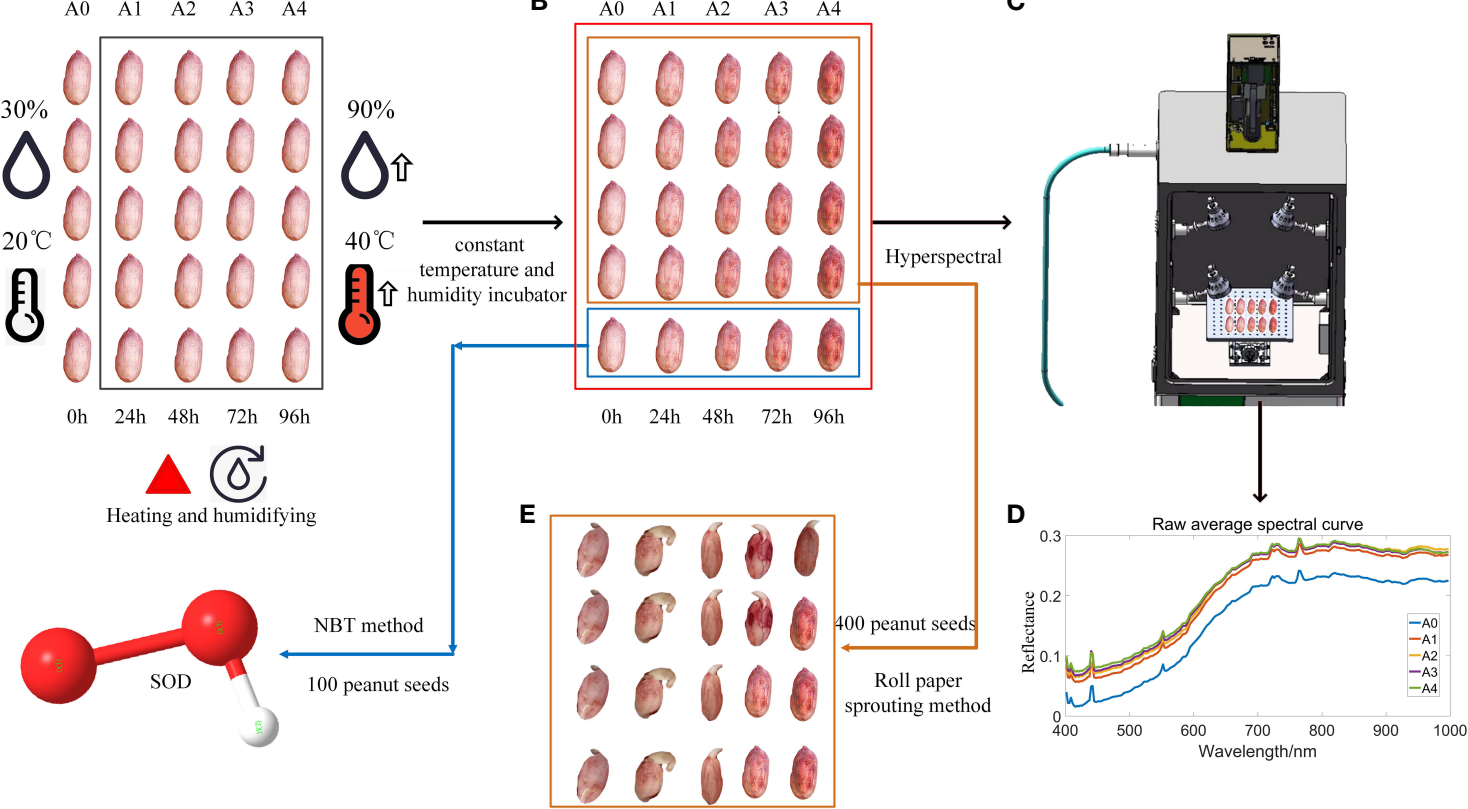
Flow chart of raw material processing steps: **(A)** Peanut aging treatment; **(B)** Two types of germination and SOD determination; **(C)** Hyperspectral determination; **(D)** Spectral data analysis; **(E)** Roll paper germination test; **(F)** Peanut superoxide dismutase enzyme activity assay.

The process of raw material processing is shown in **Figure 1**. **Figure 1A** shows that all peanut seeds are divided into 5 types of peanut original seed treatments after removing the factors such as damage and mildew, namely A0~A4, A0 peanut seeds represent fresh and unaged peanut seeds, which are cultivated in an environment with a temperature of 30°C and a humidity of 30%. At the same time, the peanut seeds of groups A1~A4 were artificially aged and placed in a constant temperature and humidity incubator with a temperature of 40°C and a humidity of 90% to simulate natural aging. Among them, the aging time of peanut seeds in groups A1~A4 ranged from 24h to 96h. As shown in **Figure 1B**, the peanut seeds obtained after treatment were divided into two groups, and 400 peanut seeds in one group were used for the roll paper germination test, which was used for the establishment of the subsequent peanut seed vigor classification model. Another group of 100 peanut seeds was used for the determination of superoxide dismutase value and used for the establishment of regression model of peanut seed superoxide dismutase value. Collect hyperspectral images of all peanut seeds to obtain their spectral data information, as shown in **Figure 1C**, and obtain the spectral curve of peanut seeds, as shown in **Figure 1D**. The peanut seeds tested in the roll paper germination test are shown in **Figure 1E**, and the relevant indicators of seed vigor were recorded, including germination rate, germination potential, germination index, average germination time, vitality index, and simple vitality index. As shown in **Figure 1F**, the SOD value was measured and its data information was recorded. Finally, the correlation analysis of the average spectral reflectance value, germination rate, germination potential, germination index, average germination time, vigor index, simple vigor index, and the average value of superoxide dismutase in each group of A0~A4 groups was established.

### 2.1 Sample preparation

In order to expand peanut seed samples with different vigor, it is necessary to perform aging of peanut seeds. Studies have shown that the overall metabolic pathways of natural aging and artificial aging were similar (Xu et al., 2013), and there was no significant difference in the aging mechanism. Therefore, artificial aging was used to treat peanut seeds. 2.5 kg of Sichuan Tianfu peanuts were purchased in the year of 2021. The peanut seeds with no mildew and damage were selected, and were divided into 5 equal parts. Each part of 0.5 kg was evenly spread in the storage tray. One group of peanuts was used as the reference group and placed at room temperature (temperature of 20°C, relative humidity of 30%) as unaged peanut seeds (A0), while the other 4 groups of peanuts were placed in an intelligent constant temperature and humidity incubator with a constant temperature. The humidity of the constant temperature and humidity incubator was set to 90%, and the temperature was set to 40°C. On the first day, the first group of peanuts was placed in the incubator and labeled as aging treatment 4d (A4). On the second day, the second group of peanuts was placed in the incubator and labeled as aging treatment 3d (A3). On the third day, the third group of peanuts was placed in the incubator and labeled as aging treatment 2d (A2). On the fourth day, the fourth group of peanuts was placed in the incubator and labeled as aging treatment 1d (A1). When the aging treatment reached the fifth day, all peanuts were uniformly taken out and placed at room temperature for 2 days to make their moisture content reach a similar level, and then placed in a low temperature box of use.

### 2.2 Hyperspectral imaging system and analysis and processing

The hyperspectral system tested is the GaiaSorter Hyperspectral Sorter from Zolix Co., ltd. (Beijing, China). And integrated Imspector series of Imaging spectrometers from Spectral Imaging Ltd. (Oulu, Finnish). The hyperspectral camera model is Gaiafluo-VN-HR(Zolix Co., ltd., Beijing, China), the spectrometer adopts a transmission grating (PGP) structure, the spectral sampling rate is 0.6 nm, and the scanning mode is built-in push-broom. The spectral range was 384-1034nm, the spectral resolution was 2.8nm, the spectral collection point was 0.65nm, the effective slit length was 8.7mm, the relative aperture was F/2.4, the slit width was 30um, A/D The output is 12, the exposure time range is 0.01-10000ms, the camera pixel is 1344×1024, the pixel size is 6.45×6.45um, the imager power consumption is 8V·A, the rated power of halogen light source is 200W, and the scanning stroke of the sample station is 400mm. The control software name is SpecView (SpecView ltd., Uckfield, UK). Hyperspectral equipment core components included a uniform light source, a spectral camera, an electronically controlled mobile platform (or conveyor belt), a computer and control software. Its working principle was to illuminate the object to be measured (sample) placed on the electronically controlled moving platform (or conveyor belt) through the light source, and the emitted light of the sample was captured by the spectral camera through the lens, and an one-dimensional image and spectral information were obtained. The moving platform (or conveyor belt) drove the sample to run continuously, so that continuous one-dimensional images and real-time spectral information can be obtained. All data were recorded by computer software to obtain a three-dimensional data cube containing image information and spectral information. The hyperspectral instrument is located in an open room of 15 square meters, Before the experiment starts, the curtains in the room are drawn to block all external light, the halogen light source inside the hyperspectral instrument is turned on, and the brightness of the indoor halogen light source is adjusted, and after the hyperspectral image is not saturated and distorted, the official collection of hyperspectral data collection information begins.

Before the image acquisition experiment, the instrument was warmed up for 30 mins, and then the instrument was adjusted and the exposure time of the camera was determined to be 10 ms, and the moving speed of the displacement platform was 1.5 mm/s. The peanut seed samples were placed on the black cardboard of the sample tray in 4 rows and 5 columns. After movement towards the electronically controlled platform, the hyperspectral imaging instrument captured the hyperspectral image of the sample and transmitted it to the computer for storage, and scanned each aging process. Gradient peanut seeds were 100, and a total of 500 seed samples were scanned. After the acquisition was completed, the original hyperspectral image was subjected to black and white correction processing to remove the dark current noise introduced in camera. The formula for black and white correction is as follows:


(1) $$R_{ci}=\;\frac{Sample_{ci}-dark_{ci}}{White_{ci}-dark_{ci}}$$

In the formula, Sample_ci_ is the original sample data, dark_ci_ is the dark background data, White_ci_ is the whiteboard data, and R_ci_ is the corrected sample data.

### 2.3 Standard germination test

Two layers of wet filter paper were spread in the germination box, 80 peanut seeds were selected from each group of the collected seeds, and placed them in the germination box in the order of spectrum collection according to the arrangement of 10 × 8 in each group. After being sprayed with a small amount of water, the box was closed and placed in an intelligent constant temperature and humidity box, under light (28°C, 8 h) and dark (20°C, 16 h) conditions, and then taken out and germinated every day. The seeds were sprayed and watered, and the relevant vigor indexes were recorded.

The remaining 100 peanut seeds were subjected to SOD superoxide dismutase physical and chemical index test. The relevant calculation formula of the vitality index is as follows:


(2) $$\text{Germination potential(GP)}=\frac{{\text{m}}_{1}}{\text{M}}\times 100\%$$

(3) $$\text{Germination rate(GE)}=\frac{{\text{m}}_{2}}{\text{M}}\times 100\%$$

(4) $$\text{Germination index(GI)}=\sum \frac{\text{Gt}}{\text{Dt}}$$

(5) $$\text{Mean germination time(MGT)}=\frac{\sum \text{Gt}\times \text{Dt}}{\sum \text{Gt}}$$

(6) Vitality index(VI)=GI×S

(7) Simple vitality index(SVI)=GP×S

In the formula, m_1_ is the number of normally germinated seeds within 3d; m_2_ is the number of normally germinated seeds within 8d; M is the total number of test seeds; Dt is the number of days of germination; Gt is the number of germinated seeds per day corresponding to the end of germination; average seedling length (cm); S is the root weight after 8 days of germinated peanuts.

### 2.4 Superoxide dismutase(SOD) activity assay

SOD activity was closely related to peanut seed vigor and played a key role in its subsequent germination. 20 peanut seeds of 5 aging groups were randomly sampled for SOD activity determination. The method of determination was nitroblue tetrazolium (NBT) method (Kono, 2022). 1 g of peanut seeds was taken, water was absorbed on filter paper, and then put it in a mortar, 5 ml of pre-cooled phosphate buffer was added, and fully ground into a homogenate on an ice bath, then the homogenate was poured into a 10 mL centrifuge tube, which was shaken for homogenization, and frozen and centrifuged at 4°C and 12000 rpm for 15min with a high-speed centrifuge. The supernatant was the crude SOD extract. The supernatant was poured into a test tube and stored at 0°C-4°C for later use. After mixing the solutions, one control group was placed under dark conditions, while the other groups were placed under 4000 Lux light, and reacted at 20°C for 10-20 min. After the reaction, a small amount of samples were taken in a 96-well microplate, and the absorbance at 560 nm was measured with a multi-function microplate reader Spark10M (A) (Hou et al., 2019).

The formula for calculating SOD (superoxide dismutase) activity (Zhao, 2017) is as follows:


(8) $$\text{SOD activity value}\frac{\text{U}}{\text{gFW}}=\frac{\left[(A_{0}-A_{b}\right)-\left[(A_{s}-A_{b}\right)]\times VT}{\frac{1}{2}\times (A_{0}-A_{b})\times W\times V_{s}}$$

In the formula: *A_b_* is the absorbance value of the dark control; *A*_0_ is the absorbance value under light; *A_s_* is the absorbance value of rice seeds; VT is the total volume of enzyme solution (mL); *V_s_* is the volume of enzyme solution used for measurement (ml); W is the fresh weight of the sample.

### 2.5 Modeling method

**Figure 2:**
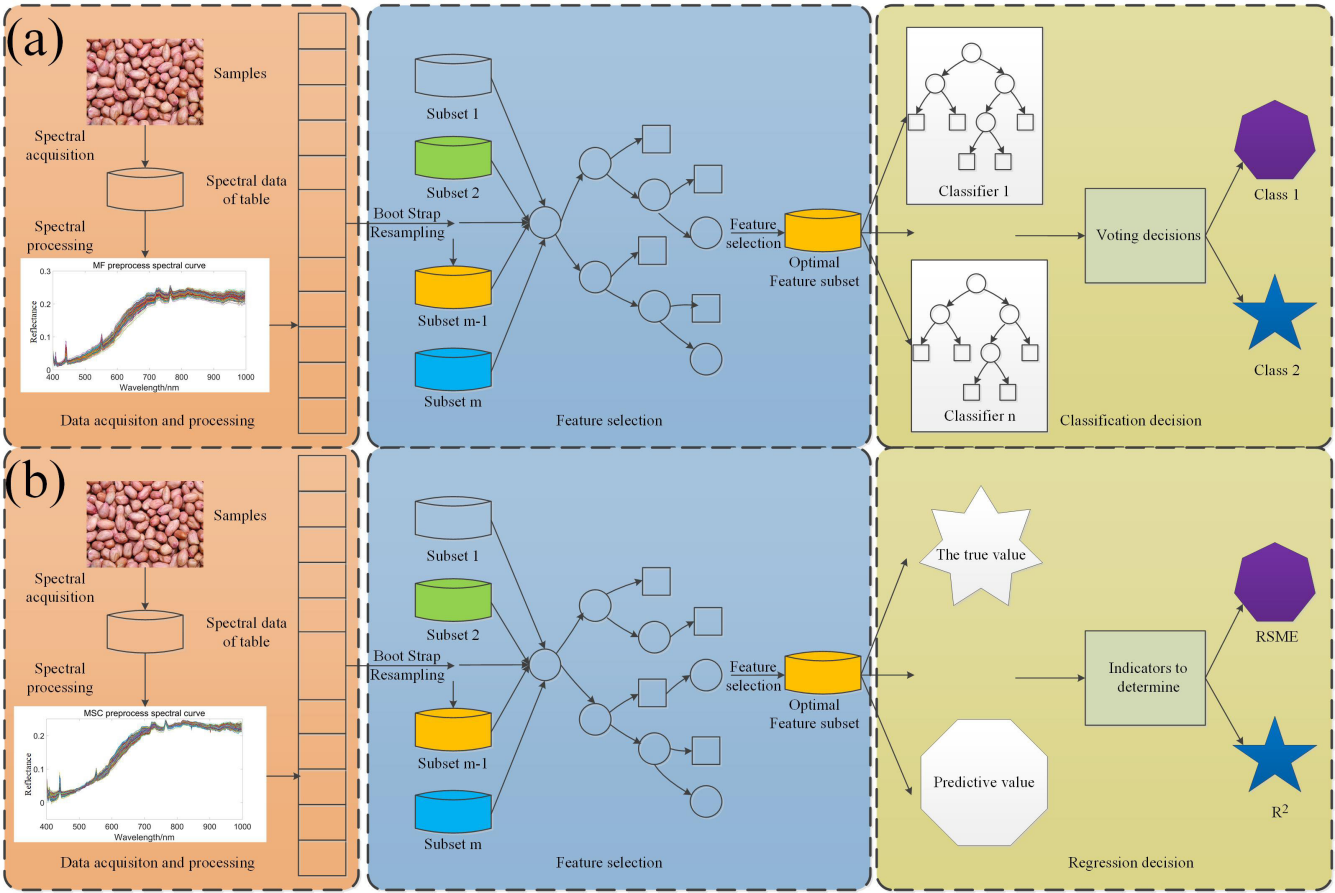
Algorithm flow chart: **(A)** Peanut seed viability classification model; **(B)** SOD regression model.

Hyperspectral seed vigor classification algorithm mainly uses Extreme gradient boosting(XGBoost), Light Gradient Boosting Machine(LightGBM), Support vector machine(SVM), Random forest(RF); SOD prediction algorithm adopts Partial least squares regression(PLSR) and XGBoost (Chen et al., 2016). XGBoost is an iterative tree-like algorithm that combines multiple weak classifiers together into a strong classifier, which is an implementation of Gradient Boosting Decision Number (Zhang et al., 2021). LightGBM (Ke et al., 2017) is an iterative boosting system, which is an improved variant of gradient decision tree (GBDT). The gradient boosting decision tree in the LightGBM algorithm is obtained by multiple iterations of the given training data set, and in each iteration, a new tree is refitted with the gradient information to join the previous iteration tree, and this in the function space can be regarded as an iterative linear combination process (Wang & Wang, 2020). SVM (Cortes & Vapnik, 1995) constructs the hyperplane as the decision surface and maximizes the isolation edge between the two classes in the classification process (Yang & Gao, 2020). The kernel function used in this study is the radial basis function. RF (Chen & Ishwaran, 2012) classifier is an ensemble classifier model composed of many decision tree classification models. RF classifier is insensitive to parameters, not easy to over fit, and has a fast training speed, which is more suitable for multi-classification problems (Wang & Chen, 2020). PLSR (Deal, 2005) is a new multivariate statistical data analysis method. It mainly studies the regression modeling of multiple dependent variables to multiple independent variables (Burnett et al., 2021).The model structure is shown in **Figure 2**.

### 2.6 Spectral preprocessing methods

In the acquisition of raw spectral data, it not only contains useful information, but also is interfered by stray light, instrument noise, sample background, baseline drift and other factors, all of which affect the selection of characteristic wavelengths, thereby affecting the quantitative and qualitative analysis results of the spectrum. Therefore, in order to make the extracted spectral information more accurately reflect the change of the sample curve, it is necessary to preprocess the original spectrum to eliminate or reduce the influence of light intensity, environmental factors and noise interference on the spectral information as much as possible. In this experiment, the Savitzky-Golay(SG) (Wang et al., 2021), multivariate scatter correction(MSC) (Liang et al., 2018) and median filter(MF) (Adams, 2021) methods were used to preprocess the original data.

### 2.7 Feature band extraction methods

The spectral data collected by hyperspectral had a large number of bands, high in-formation redundancy, large space required for data storage, long processing time, and was prone to the phenomenon of dimensional disaster, that was, the classification accuracy was reduced. Therefore, it is necessary to extract spectral data below full wavelength. The characteristic wavelengths with strong correlation with peanut seed vigor index were obtained. In this paper, CatBoost (Bentejac et al., 2021) and GBDT (Zhou et al., 2020) were used to select characteristic wavelengths, and the characteristic wavelengths with the top 15 weights were extracted to simplify the establishment of subsequent models and reduce the amount of calculation.

## 3 Results and discussion

### 3.1 Standard germination results

After 8 days of germination and culture, the test results of the vitality index were as shown in **Table 1**.

**Table 1:** Seed vigor index test result table.

aging group	total number of samples	seed vigor	seedless vitality	GP	GE	GI	MGT	VI	SVI
A0	80	77	3	96.25%	96.25%	25.66	2.78	43.37	1.63
A1	80	68	12	76.25%	85%	21.20	3.24	37.52	1.50
A2	80	52	28	53.75%	65%	15.62	4.30	24.68	1.03
A3	80	31	49	28.75%	38.75%	8.95	5.74	14.05	0.61
A4	80	18	62	11.25%	22.5%	3.17	7.21	4.82	0.32

A0, Unaged peanuts; A1, Peanuts aged 24 hours; A2, Peanuts aged 48 hours; A3, Peanuts aged 72 hours; A4, Peanuts aged 96 hours; GP, germination potential; GE, germination rate; GI, germination index; MGT, average germination time; VI, vitality index; SVI, simple vitality index.

The results in **Table 1** show that, with increase of aging time, the germination potential, germination rate, vigor index and simple vigor index of peanut seeds de-creased significantly, and the average germination time increased, indicating that the aging treatment changed the relevant physical and chemical indexes of seed vigor, thus affecting the vigor of peanut seeds.

### 3.2 Superoxide dismutase(SOD) activity results

**Figure 3:**
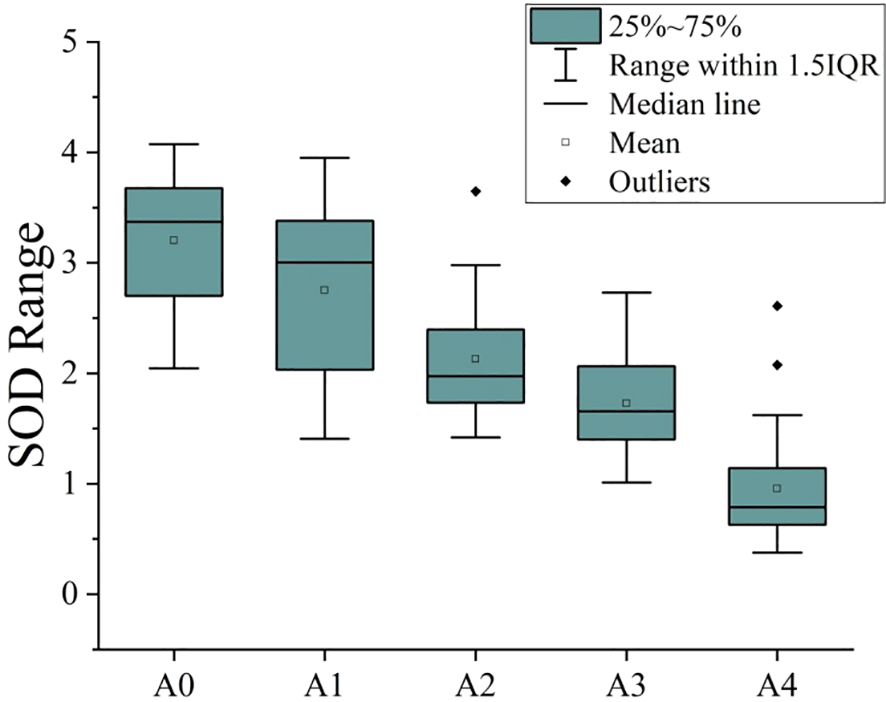
Boxplot of SOD values of peanuts with different aging degrees.

**Figure 3** is Boxplot of SOD values of peanuts with different aging degrees. The overall trend that the SOD activity value of aged peanut seeds was significantly lower than that of unaged peanut seeds. The average SOD activity value of unaged peanut seeds is above 3, and the average SOD activity value of aged peanut seeds is below 3, and with the increase of aging time, the SOD activity value is lower.

### 3.3 Spectral description

**Figure 4:**
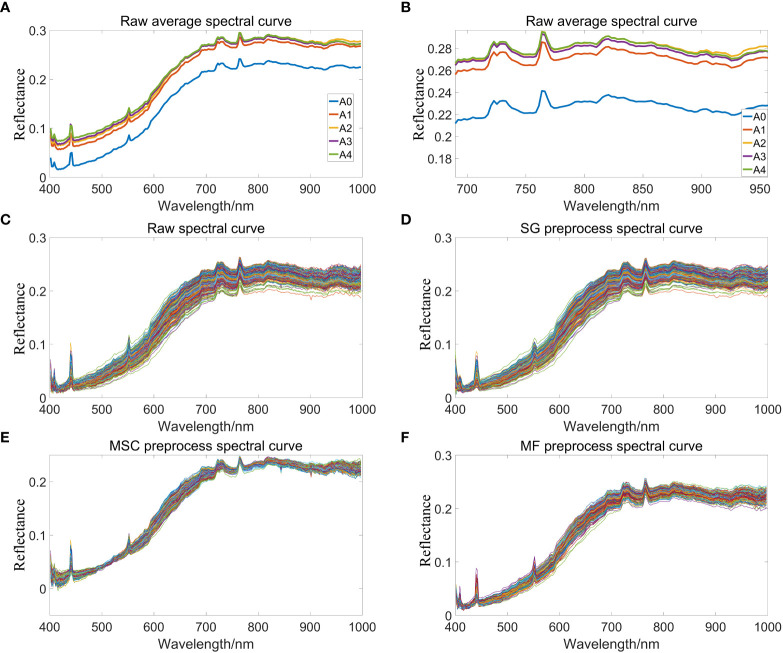
Spectral reflectance curve of peanuts. **(A)** Raw average spectral curves of peanut seeds with different aging gradients; **(B)** Partial enlarged view of spectral reflectance curve; **(C)** Raw spectral curve of peanut; **(D)** SG preprocess spectral curve of peanut; **(E)** MSC preprocess spectral curve of peanut; **(F)** MF preprocess spectral curve of peanut.

In the full band range of 384-1034nm, a large amount of noise in the band range of 384-400nm and 1000nm-1034nm will directly affect the subsequent model establishment results. After removing the head-to-tail noise bands, spectral data of 400-1000nm will be retained for curve drawing. **Figure 4A** indicate that the raw average spectral curve of peanut seeds show an upward trend, which may be due to the fact that aging accelerated the degradation of the internal storage substances of peanut seeds and reduced the content of organic matter, thereby increasing the spectral reflectance. In the spectral band of 400 nm-700 nm, the spectral reflectance of peanut seeds have a certain separability, which is due to the difference in the content of pigments and organic matter inside the peanut seeds; in the spectral band of 700 nm-1000 nm, the spectral reflectance have obvious differences mainly due to cause by the organic chemical bonds of peanut seeds. **Figure 4B** is a partial enlargement of **Figure 4A**. **Figure 4B** shows there is an obvious difference in spectral reflectance between aged and unaged peanuts, but there is little difference in the spectrum of aged peanut seeds. The peak at 450 nm is due to the strong light reflectivity of starch at 455 nm, 465 nm, and 495 nm (Wang & Wang, 2022). There is a peak change at 550 nm, which is due to the distribution of the characteristic peaks of soluble sugars (Sen, 2016) here. The spectral change around 920 nm is related to the absorption of protein. With the deepening of aging, the protein content in peanut seeds decreased, resulting in the increase of spectral reflectance. The spectral changes around 970 nm corresponded to the secondary ubiquitination stretching of the O-H bond (Raj et al., 2021) and the tertiary stretching of the C-H bond (Choi et al., 2021), causing the lipid peroxide reaction to occur inside the peanut seeds, resulting in the decomposition of organic matter into CO_2_ and H_2_O (Kou et al., 2022). **Figure 4C** is the original absorption spectrum extracted from the hyperspectral images of all peanut seeds with different aging gradients, all spectra have the same trend, indicating that peanut seeds have the same absorption characteristics at full wavelengths, five main absorption peaks are caused by the function of O—H (Liu et al., 2014), C—H (Pjw & Sk, 2016) and other functional groups. However, a large amount of useful information is masked by the irregular appearance of peanuts, and it is necessary to use spectral preprocessing methods to improve the signal-to-noise ratio of the model and display more information. **Figure 4D** shows the spectrum curve of peanuts after Savitzky-Golay preprocessing. It can be seen that the preprocessed spectrum becomes smoother than the original spectrum curve, which eliminates part of the noise, which is conducive to subsequent modeling and analysis. **Figure 4E** is the peanut spectrum curve after multivariate scatter correction preprocessing. It can be seen that the preprocessed spectrum becomes more shrunk. This is because the multivariate scatter correction can effectively eliminate the spectral differences caused by different scattering levels, and correct the baseline shift and offset phenomenon of spectral data, thereby enhancing the correlation between spectra and data. **Figure 4F** is the peanut spectrum curve after preprocessing by median filter. The peaks in the spectrum data tend to be flat, and the spectrum curve is optimized, so that the position where the spectrum curve transitions from the peak to the smooth band can better perform baseline simulation. The fitted baseline changes more gently at this position, which can effectively reduce the occurrence of under-fitting.

### 3.4 Seed vigor classification model results

If the length of the germ exceeds 1.5 cm within 8 days of the specified cultivation time, it is judged that the seed is germinating, that is, it has vigor. Otherwise, it is judged that the seed has lost its vigor, and 400 peanut seeds germinated and cultivated with rolled paper were randomly selected according to the ratio of 7:3. They were divided into the training set and the prediction set to train the model and test the performance of the model. There were 280 peanut seeds in the training set, including 173 peanut seeds with seed vigor, 107 peanut seeds without seed vigor, and 120 peanut seeds in the prediction set, including 73 seed vigor peanut seeds and 47 peanut seeds without seed vigor. The model classification results are as shown in **Table 2**.

**Table 2:** Seed vitality classification results table.

		XGBoost			LightGBM			SVM			Random Forest		
		Full	Sel1	Sel2	Full	Sel1	Sel2	Full	Sel1	Sel2	Full	Sel1	Sel2
Raw	Cal	77.50	78.33	85.83	80.83	82.50	81.66	61.79	62.14	65.83	76.07	78.21	77.50
	Pre	76.78	75.71	80.00	78.21	79.64	79.64	58.33	60.83	61.42	73.57	73.93	75.00
SG	Cal	77.5	75.83	85.83	80.83	82.50	81.66	61.79	62.14	65.83	80.00	82.5	80.83
	Pre	76.78	75.71	80.00	78.21	79.64	79.64	58.33	60.83	58.33	76.07	78.21	79.64
MSC	Cal	85.00	88.33	86.66	83.33	85.83	86.66	61.79	62.14	62.50	79.29	83.33	80.83
	Pre	80.36	84.29	84.64	76.43	82.85	75.71	58.33	60.83	59.16	76.67	81.79	79.16
MF	Cal	84.64	90.00	87.14	88.57	91.07	89.16	61.78	62.14	65.83	84.64	85.83	86.66
	Pre	82.50	87.14	88.92	87.14	90.83	87.14	58.33	60.83	62.14	80.71	82.85	86.07

Cal, calibration; Pre, prediction; Raw , raw data; Full , full wavelengths; Sel1 , selected wavelengths by CatBoost; Sel2 , selected wavelengths by GBDT; SG , Savitzky-Golay; MSC , multivariate scatter correction; MF , median filter.

**Figure 5:**
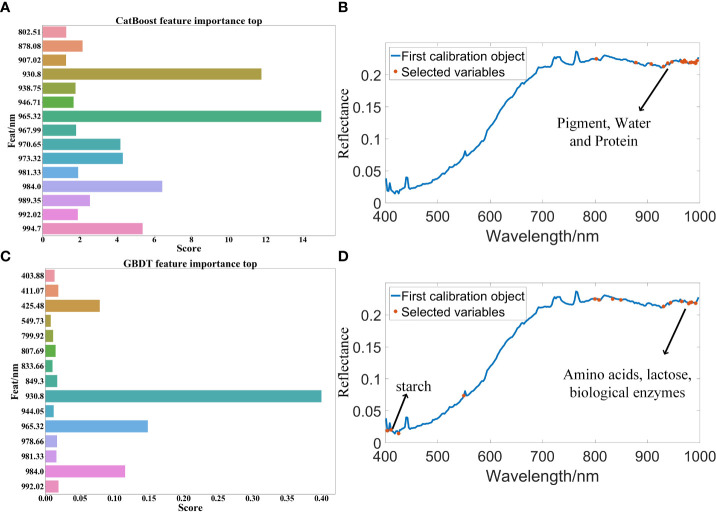
Classification model feature band extraction map: **(A)** CatBoost feature band extraction weight map; **(B)** CatBoost feature band extraction in all bands; **(C)** GBDT feature band extraction weight map; **(D)** GBDT feature band extraction in all bands.

**Figure 6:**
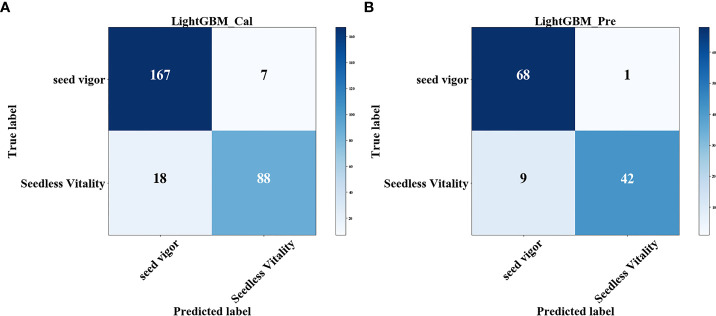
MF-CatBoost-LightGBM confusion matrix: **(A)** Training set confusion matrix; **(B)** prediction set confusion matrix.

After the feature band extraction, the classification accuracy of the modeling results is generally improved. MF is the abbreviation of median filter preprocessing method. In the optimal model MF-CatBoost-LightGBM for CatBoost feature band extraction, the feature wavelengths extracted by CatBoost included 802.51 nm, 878.08 nm, 907.02 nm, 930.8 nm, 938.75 nm, 946.71 nm, 965.32 nm, 967.99 nm, 970.65 nm, 973.32 nm, 981.33 nm, 984.0 nm, 989.35 nm, 992.02 nm, and 994.7 nm. The characteristic wavelength distribution mainly exist in the near-infrared region of 900 nm-1000 nm, because there is a certain correlation between the spectral reflectance and pigment, water and protein in this band region (Liu et al., 2014) as shown in **Figure 5D**. This result showed that this band region contained more feature information, and the correlation with peanut seed vigor is strong. **Table 2** shows that after the feature band extraction of CatBoost method, the accuracy of modeling and classification is generally improved within 5%. The weight map of CatBoost feature band is as shown in **Figure 5A**. The characteristic wavelengths extracted by GBDT in the optimal model MF-GBDT-XGBoost for GBDT feature band extraction included 403.88 nm, 411.07 nm, 425.48 nm, 549.73 nm, 799.92 nm, 807.69 nm, 833.66 nm, 849.3 nm, 930.8 nm, 944.05 nm, 965.32 nm, 978.66 nm, 984.0 nm, 981.33 nm and, 992.02 nm. The characteristic wavelength distribution is mainly around 430 nm and 900-1000 nm. This is because starch have a strong reflectivity near the 450nm wavelength (G.-l. Wang et al., 2021), and amino acids, lactose, biological enzymes and other substances have a strong reflectivity in the 900-1030 nm band (Pan et al., 2019), which shows that these substances and bands are strongly related to the vigor of peanut seeds as shown in **Figure 5B**. After the feature bands are extracted by GBDT method, the accuracy of modeling and classification is generally improved within the range of 1%-10%. The weights of GBDT feature bands are as shown in **Figure 5C**. However, **Table 2** shows that there are still some models due to too few modeling bands after the feature band extraction, and the classification accuracy is reduced. The least modeling effect is with the SVM model. The classification accuracy is between 50% and 70%. The classification effects of XGBoost, LightGBM and Random Forest are all suitable, and the classification accuracy is about 80%. Among them, MF-CatBoost-LightGBM built the model performs the best, with a classification result of 91.07% for the prediction set and 90.83% for the prediction set. MF-CatBoost-LightGBM training set confusion matrix and prediction set confusion matrix are as shown in **Figure 6**. **Figure 6** shows the classification effect of the MF-CatBoost-LightGBM model on peanut seed viability. In the training set, 280 peanut seeds, of which 255 peanut seeds were correctly classified, and in the prediction set, 120 peanut seeds, of which 110 peanut seeds were classified correctly. The results show that hyperspectral technology combined with MF-CatBoost-LightGBM model has high classification performance for peanut seed vigor.

### 3.5 Prediction result of superoxide dismutase activity value

**Figure 7:**
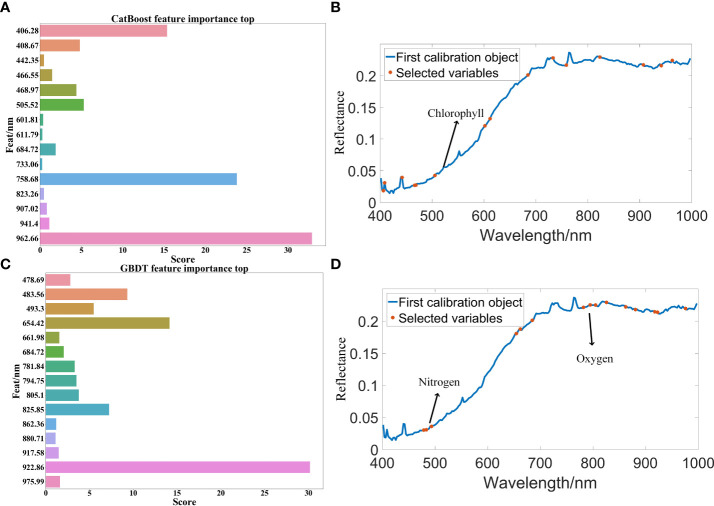
Regression model feature band extraction map: **(A)** CatBoost feature band extraction weight map; **(B)** CatBoost feature band extraction in all bands; **(C)** GBDT feature band extraction weight map; **(D)** GBDT feature band extraction in all bands.

**Figure 8:**
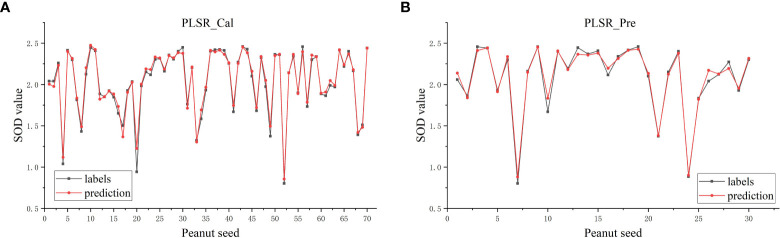
MSC- CatBoost-PLSR SOD activity prediction result graph: **(A)** Training set; **(B)** prediction set.

After the SOD activity of 100 peanut seeds was measured, SG(Savitzky-Golay), MSC(multivariate scatter correction) and MF(median filter) were used to preprocess the raw data, CatBoost and GBDT were used to extract characteristic bands from the original bands, and PLSR and XGBoost regression algorithms were used to predict SOD activity. The SOD activity prediction results are as shown in **Table 3**. The results in **Table 3** show that the data modeling effect after MSC preprocessing is significantly improved. After preprocessing, the R^2^ is about 90%, and the RMSE value is also significantly reduced. The optimal model is extracted from the CatBoost feature band. In MSC-CatBoost-PLSR, the extracted 15 characteristic bands are 406.28 nm, 408.67 nm, 442.35 nm, 466.55 nm, 468.97 nm, 505.52 nm, 601.81 nm, 611.79 nm, 684.72 nm, 733.06 nm, 758.68 nm, 823.26 nm, 907.02 nm, 941.4 nm, and 962.66 nm, the weight map of CatBoost feature band extraction is as shown in **Figure 7A**. **Figure 7B** shows the full-band distribution of CatBoost feature band extraction, and the bands near 530 nm are related to chlorophyll content. In the optimal model MSC-GBDT-PLSR for GBDT feature band extraction, the extracted 15 feature bands are 478.69 nm, 483.56 nm, 493.3 nm, 654.42 nm, 661.98 nm, 684.72 nm, 781.84 nm, 794.75 nm, 805.1 nm, 825.85 nm, 862.36 nm, 880.71 nm, 917.58 nm, 922.86 nm, and 975.99 nm, the GBDT feature band extraction weight map is as shown in **Figure 7C**, and the characteristic areas are mainly concentrated in the vicinity of 400 nm and 900 nm, indicating that the SOD activity of peanut seeds is related to related substances in this band, such as starch, protein and other substances. **Figure 7D** is the characteristic band distribution map of GBDT, the vicinity of 760 nm is related to the oxygen content (Yuan et al., 2022), and 470 nm-490 nm is related to the nitrogen content (Li et al., 2022). Among them, the optimal model MSC-CatBoost-PLSR training set R^2^ is 98.34%, RMSE value is 5.41%. The training set SOD activity prediction result is as shown in **Figure 8A**, the prediction set R^2^ is 97.87%, RMSE value is 5.66%. The SOD activity prediction result of the prediction set is as shown in **Figure 8B**, which shows that the hyperspectral imaging system could accurately predict the SOD activity of peanut seeds. **Figure 7** shows that the maximum error of the training set is lower than 0.3, and the maximum error of the prediction set is lower than 0.2. The results show that the hyperspectral technology combined with the MSC-CatBoost-PLSR model has a high prediction performance for the SOD activity value of peanut seeds.

**Table 3:** SOD activity prediction result table.

		PLSR						XGBoost					
		Full.		Sel1.		Sel2.		Full.		Sel1.		Sel2.	
		R^2^	RMSE	R^2^	RMSE	R^2^	RMSE	R^2^	RMSE	R^2^	RMSE	R^2^	RMSE
Raw	Cal	0.5942	0.6622	0.6433	0.5817	0.6358	0.5645	0.5927	0.5355	0.7107	0.5278	0.6491	0.4630
	Pre	0.5366	0.6397	0.6062	0.6260	0.6073	0.5862	0.5322	0.6189	0.5935	0.6147	0.5549	0.5899
SG	Cal	0.4875	0.6820	0.5991	0.5842	0.5839	0.6873	0.4716	0.6376	0.5270	0.6726	0.6343	0.5738
	Pre	0.4510	0.7112	0.5313	0.5849	0.5580	0.5679	0.4278	0.7138	0.4278	0.7138	0.4278	0.7138
MSC	Cal	0.9040	0.1309	0.9834	0.0541	0.9769	0.0676	0.9658	0.0736	0.9724	0.0666	0.9592	0.0804
	Pre	0.8701	0.1483	0.9787	0.0566	0.9659	0.0755	0.9542	0.0852	0.9563	0.0825	0.9247	0.1094
MF	Cal	0.6474	0.5272	0.6428	0.5528	0.6890	0.5701	0.7352	0.4853	0.7368	0.4838	0.7809	0.4414
	Pre	0.5302	0.6447	0.6358	0.5645	0.6881	0.5643	0.6959	0.5655	0.7009	0.5158	0.7363	0.4842

Cal, calibration; Pre, prediction; Raw, raw data; Full, full wavelengths; Sel1, selected wavelengths by CatBoost; Sel2, selected wavelengths by GBDT; R2, Correlation coefficient; RMSE, root mean square error; SG, Savitzky-Golay; MSC, multivariate scatter correction; MF, median filter.

### 3.6 Correlation analysis

**Figure 9:**
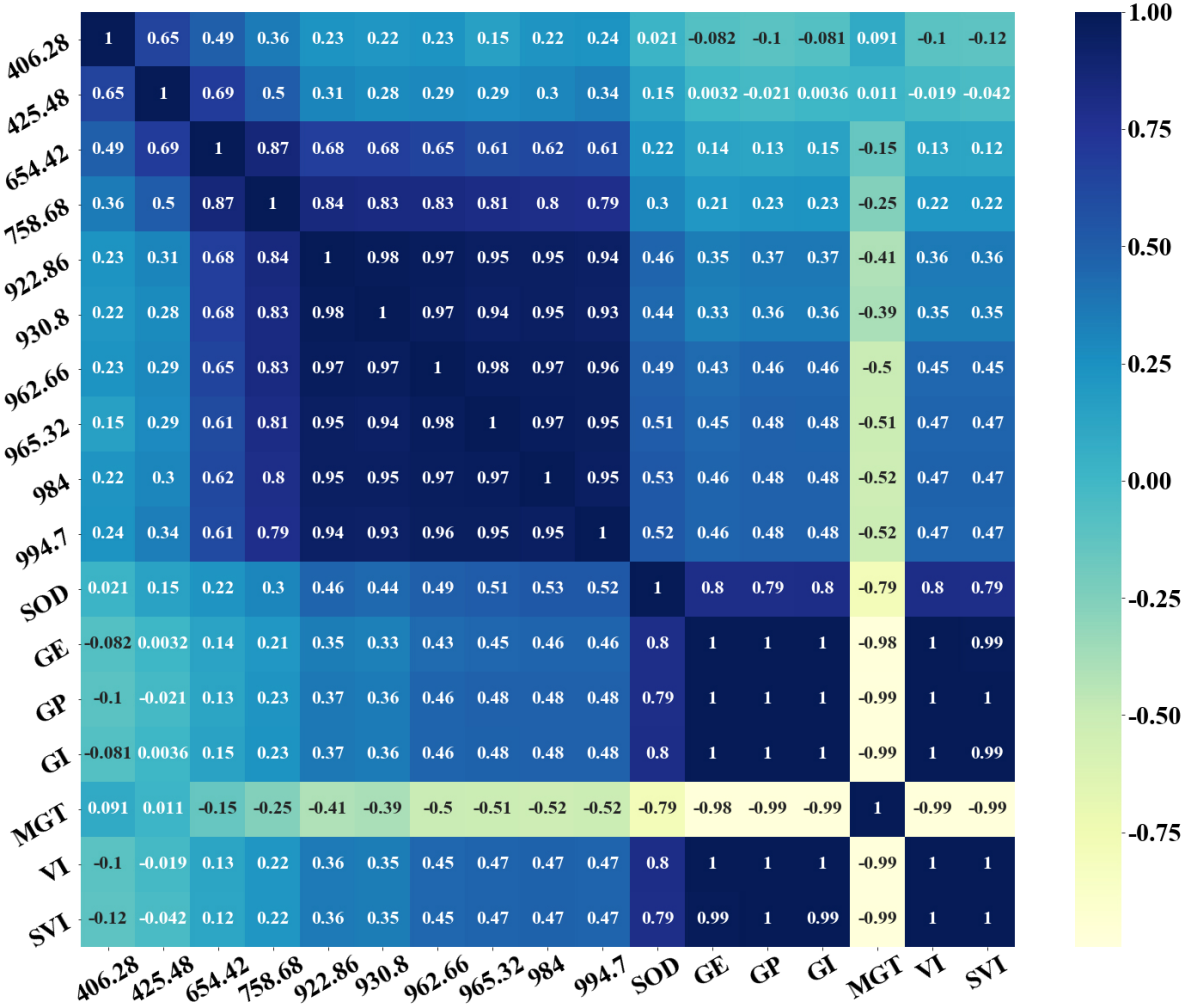
Characteristic bands, vitality indicators and SOD activity heatmap.

The top five weights of the characteristic bands of peanut seed vigor classification are 425.48nm, 930.8nm, 965.32nm, 984.0nm, and 994.7nm, respectively. The top five predicted characteristic band weights of SOD value of peanut seeds are 406.28nm, 654.42nm, 758.68nm, 922.86nm and 962.66nm respectively. The average value of spectral reflectance and the average value of SOD value of peanut seeds in each group of A0~A4 groups were calculated. The correlation analysis of peanut seed vigor index(germination rate, germination potential, germination index, average germination time, vitality index, and simple vitality index), average spectral reflectance, average SOD, top five seed vigor classification characteristic band weights, and SOD value prediction characteristic band weight top five in groups A0~A4 were established. **Figure 9** shows the heat map of the correlation analysis. It can be seen from the figure that the self-growth index of seed vigor has a high correlation, almost all reaching 1, and GE, GP, GI, VI, and SVI are positively correlated, while MGT is negatively correlated with other seed vigor indexes. This is due to the calculation formula of the seed vigor index, and the average germination time is a negative index of the seed vigor index. Generally, the higher the vigor of peanut seeds, the shorter the average germination time. The bands at 922.86nm, 930.8nm, 962.66nm, 965.32nm, 984nm and 994.7nm had strong correlation with peanut seed vigor index, and the correlation with GE was 0.35~0.46, and GP and GI was 0.37~0.48. The correlations with MGT were -0.41~-0.52, and with VI and SVI were 0.36~0.47. It is not difficult to see that most of these bands come from the characteristic bands of peanut seed vigor classification, which are consistent with the previous description of peanut seed vigor classification. All characteristic bands are also highly correlated with the SOD value of peanut seeds, up to 0.52, which is consistent with the regression analysis of peanut seeds SOD value described above. The most important thing is that the SOD value of peanut seeds has a strong correlation with the peanut vigor index, reaching 0.8, this also verifies the fact that SOD participates in the catalytic disproportionation reaction with reactive oxygen species and free radicals as substrates, and its activity level directly affects the seed vigor.

## 4 Conclusion

Seed vigor plays a crucial role in crop growth stage, and SOD enzyme activity is highly correlated with seed vigor indicators. In this paper, hyperspectral imaging technology was used to identify the vigor of peanut seeds and predict the activity of SOD inside the seeds. After the peanut seeds were germinated and cultivated by the method of rolling paper germination, the correlation analysis of characteristic bands, SOD activity and seed vigor indexes were made. After comparing and analyzing the results of various classification models, it was discovered that the MF-CatBoost-LightGBM model has the best effect and the best discrimination effect on peanut seed vigor. The accuracy rate of the training set reach 91.07% and the accuracy rate of the prediction set reach 90.83%. The MSC-CatBoost-PLSR model has the best effect when the SOD activity inside the seeds is analyzed by regression, in which the R^2^ of the training set is 0.9834, the RMSE value is 0.0541, the R^2^ of the prediction set is 0.9787, and the RMSE value is 0.0566. The correlation analysis of characteristic bands, vigor index and SOD activity shows that SOD activity of peanut seeds has strong correlation with seed vigor index, the correlation coefficient between the vigor index and SOD value reaches 0.8. In summary, the use of hyperspectral imaging technology can accurately discriminate peanut seed vigor indexes, and can predict the SOD activity inside seeds. At the same time, the strong correlation between SOD activity and seed vigor indexes can provide new opportunities for future seed quality testing machinery ideas.

## Data availability statement

The original contributions presented in the study are publicly available. This data can be found here: https://github.com/cjkka/cjkka/tree/main.

## Author contributions

Conceptualization, ZZ and QiaW. Data curation, JZ and MZ. Formal analysis, YZ and QinW. Funding acquisition, YW and YC. Methodology, TL and JC. Project administration, MZ. Writing—original draft, JC and JL. Writing—review and editing, ZZ and WW. All authors contributed to the article and approved the submitted version.
